# More Questions Than Answers for Adolescents and Young Adults With Cancer

**DOI:** 10.1093/jncics/pkab040

**Published:** 2021-05-20

**Authors:** Nita L Seibel, Denise Riedel Lewis

**Affiliations:** 1 Division of Cancer Treatment and Diagnosis, Cancer Therapy Evaluation Program, Clinical Investigations Branch, National Cancer Institute, National Institutes of Health, Rockville, MD, USA; 2 Division of Cancer Control and Population Sciences, Surveillance Research Program, National Cancer Institute, National Institutes of Health, Rockville, MD, USA

Since initial recognition of the term *adolescent gap* in 1997, followed by the 2006 report from the Adolescent and Young Adult (AYA) Progress Review Group sponsored by the National Cancer Institute and LIVESTRONG Foundation, researchers have been trying to understand the issues responsible for this survival gap in AYA oncology patients (1,2). Multiple factors have been proposed to explain this gap, including one of delayed access to care resulting in advanced disease at presentation (2). Over the years, AYA researchers from the University of Southern California have added to our knowledge of AYA oncology outcomes by using the National Cancer Institute Surveillance, Epidemiology, and End Results cancer registry data. They showed that survival for AYA patients with cancer has exhibited sustained improvement and relative superiority to other age groups when HIV/AIDS-related cancers were excluded and after HIV/AIDS was effectively controlled. They also emphasized the importance of taking baseline survival into account when comparing survival improvements for time periods with other age groups (3). This same group of AYA researchers used California Cancer Registry data to identify cancers among AYA patients for which survival had improved, highlighting those cancers for which survival remains poor and the disparities that affect patients with advanced disease stage, those from racial and ethnic minority populations, and those with low socioeconomic status. Specifically, these investigators found that disease stage was the strongest predictor of mortality among AYA patients, regardless of cancer site, and that AYA patients diagnosed with metastatic cancer have a 6-fold higher mortality rate compared with patients who have localized disease (4).

In this issue of the Journal, Bhutada et al examine the incidence patterns, trends, and disparities of metastatic disease in AYA patients with cancer compared with middle-aged (40-64 years of age) and older adults (65-79 years of age) to provide further insights into the factors related to survival outcomes for AYA oncology patients and to guide diagnosis and treatment (5). Two points stand out from this study. First, using SEER18 program data from 2000 to 2016, Bhutada et al compared the incidence of patients presenting with metastatic disease and found that when compared with older adults, the proportion of AYA patients who have metastatic disease was equivalent or lower in every site except stomach cancer and breast cancer (57.3% vs 46.4% of middle-aged patients and 39.5% of older adults for stomach cancer; 6.6% vs 4.4% for middle-aged patients and 5.6% for older adults for breast cancer; 2-sided *P *<* *.001 for all comparisons) (5). These findings suggest that AYA patients are generally not more likely to present with metastatic disease than older adults with the same cancer, except for the 2 cancers noted above. So, if diagnosis is delayed, which often happens with AYA patients, it is not reflected in the stage of disease at presentation for the AYA patient. It is therefore unlikely that the current disparity in survival improvement in this age group is related to more AYA patients having metastatic disease at presentation than other adult age groups. A concern that Bhutada et al describe, however, is the increasing incidence rates for AYAs of metastatic breast, stomach, and kidney cancers and for adolescents and middle-aged adults with colorectal cancer compared with older adults (5). We have known about the aggressiveness of breast cancer in younger women, particularly non-Hispanic Black women, which has been the subject of investigations (6-9). The recognition of increased incidence rate ratios for stomach cancer and kidney cancer, particularly in non-Hispanic Black AYA patients, is new and worrisome.

The second notable point from the Bhutada et al. article is the proportion of AYA patients presenting with metastatic stomach cancer, which was statistically significantly higher than in the middle-aged and older adult groups (5). Although there have been some publications on occurrence in the Hispanic population and in younger adults (<50 years of age), this cancer has not been widely associated with the young adult population. This finding suggests a delay in diagnosis for these patients because symptoms are nonspecific and awareness of the incidence and risk of stomach cancer in AYA patients has not been appreciated (10–13). This rise in occurrence mirrors the observations of increased colorectal cancer incidence in young adults, a trend for which there is now greater awareness (14). The NCI and National Institute of Environmental Health Sciences convened a virtual meeting in September 2020 to examine evidence of potential risk factors, mechanisms, and opportunities for screening and treatment for early-onset colorectal cancer. Similar to the investigations into the increased colorectal cancer incidence in young adults, some of the same factors may apply to stomach cancer, including diet, obesity, infection, and molecular markers of exposure (15).

As a result of this study, many unanswered questions and perhaps warnings should direct future research efforts. Although poorer outcomes for AYA patients with cancer do not seem to be linked to higher incidence of metastatic disease at presentation, this trend may be changing. Why are we seeing a faster rise in the number of AYA people with metastatic breast, stomach, and kidney cancer compared with other adult age groups? Is there a relationship with some of the factors being investigated for the increase in young adult colorectal cancer that could play a role in metastatic stomach and kidney cancer in AYA patients? How do we educate providers to consider colorectal cancer, stomach cancer, and kidney cancer when seeing a young adult with nonspecific symptoms? Is there a difference in molecular biology for these cancers when they occur in young adults compared with older adults? Is there a population of young adults at risk for a specific cancer that could benefit from screening, and which screening tools could be used? Are treatment trials in metastatic breast, kidney, stomach, and colorectal cancer available for young adults? All these questions can hopefully be answered in future.

## Funding

None.

## Notes

**Role of funder:** Not applicable.

**Disclosures:** The authors have no conflicts of interest to disclose.

**Author contributions:** Both authors wrote and approved the final version of manuscript.

**Disclaimer:** The content is solely the responsibility of the authors and does not necessarily represent the official views of the National Cancer Institute or the National Institutes of Health.

## Data Availability

Not applicable.
